# The Second Life Metaverse and Its Usefulness in Medical Education After a Quarter of a Century

**DOI:** 10.2196/59005

**Published:** 2024-08-06

**Authors:** Francisco Sendra-Portero, Rocío Lorenzo-Álvarez, Teodoro Rudolphi-Solero, Miguel José Ruiz-Gómez

**Affiliations:** 1 Department of Radiology and Physical Medicine Facultad de Medicina Universidad de Málaga Málaga Spain; 2 Department of Emergency and Intensive Care Hospital de la Axarquía Vélez-Málaga Spain

**Keywords:** medical education, medical students, postgraduate, computer simulation, virtual worlds, metaverse

## Abstract

The immersive virtual world platform Second Life (SL) was conceived 25 years ago, when Philip Rosedale founded Linden Lab in 1999 with the intention of developing computing hardware that would allow people to immerse themselves in a virtual world. This initial effort was transformed 4 years later into SL, a universally accessible virtual world centered on the user, with commercial transactions and even its own virtual currency, which fully connects with the concept of the metaverse, recently repopularized after the statements of the chief executive officer of Meta (formerly Facebook) in October 2021. SL is considered the best known virtual environment among higher education professionals. This paper aimed to review medical education in the SL metaverse; its evolution; and its possibilities, limitations, and future perspectives, focusing especially on medical education experiences during undergraduate, residency, and continuing medical education. The concept of the metaverse and virtual worlds was described, making special reference to SL and its conceptual philosophy, historical evolution, and technical aspects and capabilities for higher education. A narrative review of the existing literature was performed, including at the same time a point of view from our teaching team after an uninterrupted practical experience of undergraduate and postgraduate medical education in the last 13 years with >4000 users and >10 publications on the subject. From an educational point of view, SL has the advantages of being available 24/7 and creating in the student the important feeling of “being there” and of copresence. This, together with the reproduction of the 3D world, real-time interaction, and the quality of voice communication, makes the immersive experiences unique, generating engagement and a fluid interrelation of students with each other and with their teachers. Various groups of researchers in medical education have developed experiences during these years, which have shown that courses, seminars, workshops and conferences, problem-based learning experiences, evaluations, teamwork, gamification, medical simulation, and virtual objective structured clinical examinations can be successfully carried out. Acceptance from students and faculty is generally positive, recognizing its usefulness for undergraduate medical education and continuing medical education. In the 25 years since its conception, SL has proven to be a virtual platform that connects with the concept of the metaverse, an interconnected, open, and globally accessible system that all humans can access to socialize or share products for free or using a virtual currency. SL remains active and technologically improved since its creation. It is necessary to continue carrying out educational experiences, outlining the organization, objectives, and content and measuring the actual educational impact to make SL a tool of more universal use.

## The Metaverse, Virtual Worlds, and Second Life

### The Metaverse: Concept and Evolution

The term *metaverse* appears for the first time in Neal Stephenson’s novel *Snow Crash*, published in 1992 [1], which recreates a fictional virtual world reproduced by a computer in which users interacted with each other and with the elements of the world through a representation of themselves called an avatar. Cinema has popularized other examples of virtual worlds parallel to the real one in the *Matrix* tetralogy (1999-2021), written and directed by Lana and Lilly Wachowski, or the film *Ready Player One* (2018), directed by Steven Spielberg. The truth is that the possibility of reproducing 3D virtual environments in which the user can enter and interact with everything that exists in them is technologically feasible today, largely thanks to the development of video games for consoles and computers and more recently through the dissemination of massively multiplayer online role-playing games (MMORPGs), with thousands of young, and not so young, users worldwide.

Conceptually, the metaverse is an open system, a vast virtual world that can be accessed simultaneously by millions of people through highly customizable avatars and powerful experience creation tools integrated with the offline world through its virtual economy and external technology [2]. The metaverse has been defined as an interconnected 3D virtual environment in which people from all over the world can come together to share social experiences [3], “a computer-generated universe” in which people immerse themselves in experiences rather than simply observing them [4].

**Figure 1:**
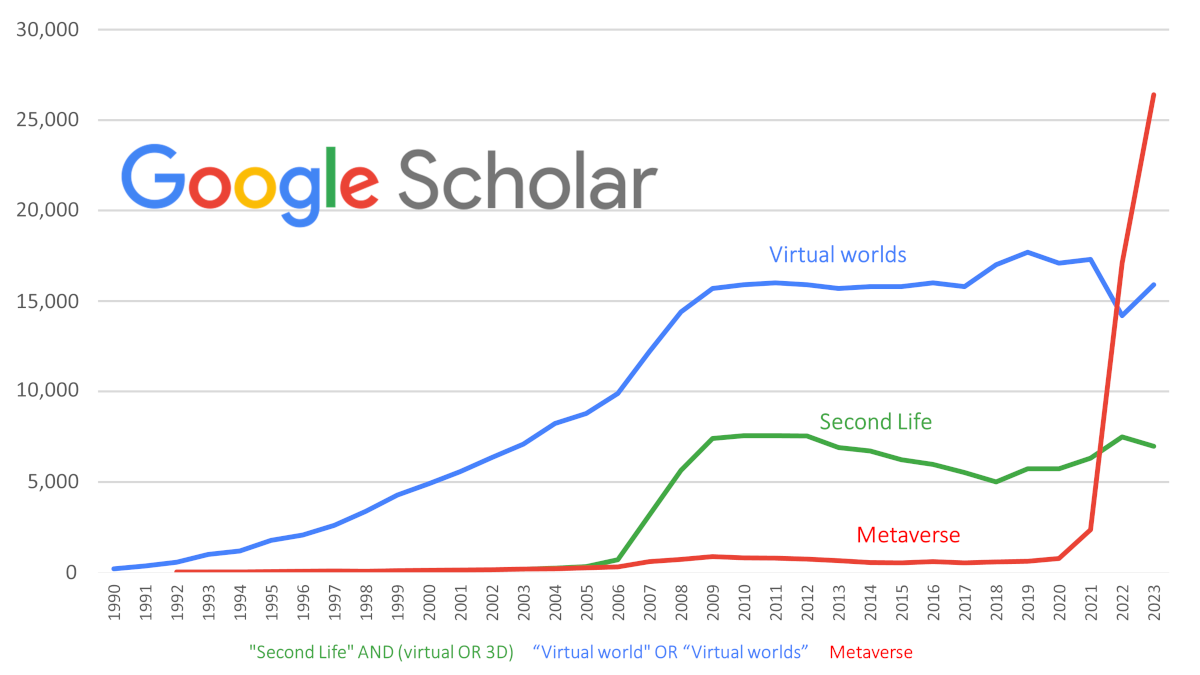
Google Scholar search graph by year for the terms virtual worlds, Second Life, and metaverse. Below the horizontal axis, the search strings used are shown.

The term *metaverse* has gained great popularity recently (Figure 1) after Mark Zuckerberg, chief executive officer of the social network Facebook, announced in October 2021 his company’s interest in developing the metaverse as a social environment, apparently as an area of interaction and commercial transactions. As a result, there is currently a growing interest in all matters related to the metaverse; virtual worlds; and their applicability to today’s society, including education.

Publications about the metaverse include various technological approaches that can lead to some confusion, such as augmented reality (AR), virtual reality (VR), extended reality (XR), or virtual worlds [3,5]. AR is a technology that increases our perception of reality by adding digital information to the real world; VR allows users to experience a completely simulated environment as if it were real; mixed reality is a hybrid of the physical and virtual worlds in which digital objects interact with the physical environment to create an even more immersive experience; and XR is a general term to refer to AR, VR, and mixed reality [5].

Some differences have been pointed out between the metaverse and XR technologies [6]. The concept of the metaverse emphasizes sharing the experience, interacting with other avatar-mediated humans; thus, a student learning by means of XR cannot be considered an example of a metaverse per se [7]. The metaverse is a new method of social connection, a virtual world in which people can autonomously participate in social activities such as meetings, discussions, collaborations, and games [8] and that provides people with unique learning opportunities [9,10]. In a similar way, virtual worlds and VR, while emerging from similar roots, are not interchangeable terms. VR requires a special interface with the electronic space—head-mounted devices with glasses and headphones—as well as hand controllers to interact within a virtual space. VR pays little attention to what is happening within the virtual world by other people or the community [11]. A VR platform can even be designed to present the context in a more visually attractive and *realistic* way to a single user, which completely distances it from the concept of a metaverse.

### Virtual Worlds

Virtual communication has become an integral part of the 21st-century culture [12]. The application of communication technologies has contributed to the development of virtual worlds where users interact without a specific script and even have the freedom to develop the contents of the world and the objectives to which they want to dedicate themselves [3]. Virtual worlds allow participants to interact with contents and visitors as much as they do in real life but without having to physically move to a common place in which to establish such interaction. The range of activities that can be developed in these environments is practically unlimited and depends fundamentally on the ability of the developers of these malleable spaces to transform them into a particular environment [13].

A virtual world is a synchronous and persistent network of people represented by avatars facilitated by networked computers [14], a shared space that hosts a web-based community [15,16]. The main characteristics of virtual worlds have been described as follows [17]: (1) a shared space in which multiple users can participate at the same time; (2) a graphical user interface, which visually represents the virtual space; (3) immediacy or real-time interaction; (4) interactivity as users have a certain degree of control over the content; (5) persistence (it is an always “active” platform); and (6) socialization and community as in-world social groups, gatherings, and neighborhoods are encouraged. A virtual world is also a 3D virtual space created by the site manager for a specific purpose (ie, a collaborative workspace, a classroom, a virtual laboratory, a playground, a discussion forum, or a meeting place with friends). Therefore, virtual worlds can become virtual academic places where users, students, and teachers enter and interact by means of an avatar, being able to speak, chat, and visualize various educational contents.

The roots of virtual worlds can be found in video games and social networks [18]. Although the development of virtual worlds has reached a high level of use through simulation games, the former should not be assimilated to games. A virtual world is not an MMORPG, that is, a “game”; however, an MMORPG can exist within a virtual world [19]. Even though many successful multiplayer games are carried out in virtual worlds, their possibilities are unlimited and not restricted to the world of gaming. In this sense, one of the activities that has been successfully tested in virtual worlds is basic education in the fields of science and arts [20-22]. Some examples of virtual worlds are Active Worlds, Sinespace, Instant Messaging Virtual Universe, OpenSimulator, Sansar, and Second Life (SL) [17]. The latter has been described as a virtual world with great educational possibilities in higher education [21,22] and is the subject of this paper.

### SL Virtual World

#### A Story of a Quarter of a Century

The immersive virtual world platform SL was conceived 25 years ago, when Philip Rosedale founded Linden Lab in 1999 with the intention of developing computing hardware that would allow people to immerse themselves in a virtual world. This initial effort was transformed 4 years later into SL, a universally accessible virtual world centered on the user, with commercial transactions and even its own virtual currency. SL is one of the most well-known and used virtual worlds, a virtual community created or developed by its own users. SL is not a game but a multiuser virtual environment; it has always tried to distance itself from the video game universe [2]. From its beginning, it has been presented as an open world with no explicit objectives, quests, or missions, explicitly and flatly stating the following: “If you can imagine it, you can do it here” or “You choose your own goals” [2].

SL experienced a huge rise in popularity. Until approximately 2008, virtual worlds such as SL were all the rage, with headlines trumpeting the life-altering potential of these avatar-occupied worlds. Philip Rosedale considered “alternative universes” such as SL a new medium of human expression that could be the “next big thing for the Internet” [17]. However, by approximately 2010, SL and other virtual worlds lost their former appeal, replicating the Gartner Hype Cycle, a graphical representation of a common pattern that emerges with each new technology or other innovations. The cycle begins with overenthusiasm followed by a period of disappointment, reaching a plateau of slow growth when the relevance and role of the innovation are finally understood. The lack of attraction among internet users could be due to several factors [17]: (1) the learning curve necessary to acquire even a modest level of competence in the virtual world; (2) time being a limiting factor, so the hours invested in “living a second life” reduce the 24 hours available for real-life activities; and (3) the technical sophistication of the platform in relation to other social interaction offerings that appeared disruptively in those years, such as Facebook, Instagram, Twitter, Snapchat, and others. In short, too many easier-to-use alternatives appeared on the scene 15 years ago. The current position of SL (and other virtual worlds) in the Hype Cycle is an open question. Several studies suggest that it is on a “productivity plateau,” which implies stable but unspectacular growth over time [17]. An internet search may give an idea of this (Figure 1).

In 2020, Linden Lab was purchased by the Waterfield Group, an investment firm that buys companies that obtains decent earnings and helps them remain profitable in perpetuity [2]. That same year, the COVID-19 pandemic put the world under quarantine, and many people spent their quarantine in virtual worlds, eager to connect with others. SL saw a surge in old and inactive users as growing awareness of virtual worlds fueled renewed and growing interest in the metaverse.

Now, 25 years after the foundation of Linden Lab, this virtual world seems to still have some weight, especially in the world of creation and graphic design [23]. In 2021, it was reported that there were still approximately 64.7 million active users on SL, and Web Tribunal stated that, by 2023, this had risen to 70 million accounts, with a daily average of 200,000 users in 200 countries [24]. SL continues to be successful as a virtual world model that connects with the concept of an equitable, diverse, and creative metaverse. Its creativity tools are open and powerful, and its user community is diverse and made up of members free to create endless values and possibilities [2].

#### What Is SL?

From a technical point of view, the SL architecture is based on a client-server model. The graphical user interface runs locally, whereas the 3D virtualization runs on servers owned by Linden Lab. The visual experience is presented in real time, but the objects are stored remotely. This makes it easier for the user to create content, but on the other hand, it puts a lot of pressure on the user’s graphical capabilities and bandwidth [25].

From the user’s experiential perspective, the visual and physical realism of the virtual space and the interaction and communication with others combine to produce a deeply immersive experience. Immersion is the sensation of being so situated within the 3D virtual world that awareness of the environment beyond the digital screen practically disappears [2]. It contributes to creating a feeling of “being there” and a strong sense of copresence when other avatars are present.

From a social point of view, SL is a virtual community created or developed by its own users. Through the avatars, one has the feeling of being part of that world and that others can perceive it at the same time, the feeling of social presence on a virtual global platform currently shared by diverse generations, including baby boomers, Millennials, and Generations X and Z [2,17]. Within SL, there are multiple communities with their own spaces that recreate scenarios designed by the users themselves. These communities have their own economy based on a virtual currency, the Linden Dollar, with which users can buy and sell virtual material, make transactions, pay for services, and exchange other currencies [26] (1 Linden Dollar is equivalent to €0.0029 or US $0.0031 as of March 27, 2024). The importance of this economic system is such that it has required the development of a virtual monetary policy that manages resources and how to carry out transactions, which can be done in a common market (“Marketplace”) or directly between users [27]. Within the communities, there are also a series of rules, which in case of noncompliance can lead to the blocking or deletion of the user account. These rules prohibit intolerance, harassment, aggression (even virtual), slander, indecency, and disturbance of the peace [28].

#### How It Works

Information related to SL can be found on its web page [29]. To access SL, the user must create an account, choose an avatar, and download the SL viewer [30] or any other SL-compatible viewer [31] to display the virtual world on their computer screen. The use of SL is free for users (although a premium account can be purchased for US $9 per month) but not for landowners and administrators, who have to pay different rates according to the size of the land.

The SL virtual world imitates the real world that we know. It consists of interlinked regions that contain land, water, and sky. A region is an entire island that is 256 × 256 m, or 65,536 m^2^. Almost all the objects that are visible in SL are built from 3D geometric primitive shapes called prims. Each region has an allotment of 15,000 prims. Prims can assume any desirable shape, and one can make prims look any way wanted by applying selected textures to their surfaces. They can be given certain qualities and features (such as transparency or the ability to ﬂex or bend with the wind), they can be linked together, and they can be made to do things through a script written in Linden Scripting Language [32]. Prims also allow for sharing media (ie, displaying a web page or a linked video on a face).

The avatar is the representation of the user inside SL, and by means of it, they can interact with the virtual world. Interactions include viewing the world with different perspective and focus, touching objects, answering to displayed menus originated from Linden Scripting Language scripts, moving or adopting gestures (walking, running, flying, teleporting, sitting, or lying down), and communicating with others (receiving and sending audio, chat, instant messages, and note cards). At launch, SL avatars were human by default but not ultrarealistic. The internal prim creation tools encouraged the construction of avatar attachments, which led to a wide variety of avatar types and creative environments to explore. The arrival of mesh in SL in 2010—high-resolution 3D files created in offline software and then uploaded into the virtual world—greatly changed this dynamic. Thanks to mesh and other graphics enhancements, SL avatars and environments now look as detailed and as vivid as those from top video games [2].

Communication between avatars can be done through voice chat, written chat, and note cards. Voice chat gives a very important sense of presence; it gives the user the perception that they are in front of other human beings and allows them to perceive the nuances of verbal communication. Its use can be public (everyone within a predetermined distance can hear it) or private (the conversation is exclusive to an avatar or group of avatars) but only alternatively [33].

Written chat can also be public (local chat) or private (instant message). Local chat can be read by everyone nearby, is great for asking short-answer questions to the audience (yes and no or true and false), and allows attendees to ask questions to the presenter without interrupting the presentation. Instant messages are sent from one avatar to another, who receives it immediately if they are logged in SL or as soon as they log in to SL. Note cards are messages that are sent in SL and are stored in the inventory of the avatar that receives it, recording the date and time of sending and avatar of origin. In educational activities, they are very useful to collect information from students as proof of their attendance or to complete a task or an exam (open answer or multiple choice), requesting that they send a note card to the teacher’s avatar [33].

In essence, through the SL environment, we can provide specific contents to users’ avatars (students, teachers, or the public in general), such as text, slide presentations, or other multimedia content. Moreover, we can also communicate verbally in a synchronous way (in real time) with other avatars, creating a meeting- or classroom-like environment in which we can give (or attend) a talk or organize a discussion group on a specific topic. This paper aims to review medical education in the SL metaverse, its evolution, its possibilities and limitations, and future perspectives. In the following sections, general aspects of SL as an educational tool are first addressed, including its contribution to higher education, and later, the contributions to medical education are analyzed.

## SL as an Educational Tool

### Learning Theories and Virtual Worlds

Learning theories are constructs that explain how human beings acquire knowledge, values, skills, behaviors, attitudes, and aptitudes in a systematic, dynamic, and progressive way through different training techniques such as study, reflection, experimentation, or teaching [34]. The most important theories currently considered are behaviorist theory, cognitivist theory, and constructivist theory, and all 3 are integrated into the use of virtual worlds such as SL [35,36]. The *behaviorist theory* focuses on the establishment of observable responses through the student’s behavior based on a series of stimuli received during learning, which makes it necessary to use small check points to achieve the behavioral objective [35]. Virtual worlds have been described as a suitable tool to effectively stimulate students to perform their tasks better. These environments, which can present a playful atmosphere, allow a series of rewards or scoring systems to be established, which motivates students to carry out their learning [37]. The *cognitivist theory* describes the mental process that takes place in the students and how they can apply what they learn in new experiences [35]. Virtual environments such as SL allow for the development of experiential experiences in which the student can carry out this transfer of knowledge. The *constructivist theory* holds students responsible for their own training, providing them with the necessary tools to create their own learning methodology, focusing on students’ relationship with each other and with educators [35]. Virtual worlds, and particularly SL, are social environments in which students can participate in activities with a constructivist approach, including learning experiences that imitate the real world, interacting live with classmates and teachers. This dynamic allows students to process the information received and construct meaning actively instead of receiving information passively [36].

Learning is based on the passage of knowledge from working memory (short term) to long-term memory [38,39]. This process is analyzed using a factor known as cognitive load, defined as the mental effort from the cognitive system necessary to perform a task [40,41]. The complexity of the tasks, as well as their organization and the presentation of information imposed on learners, can hinder the function of short-term memory in a given task. The intellectual complexity of the tasks is known as intrinsic cognitive load, whereas their presentation is known as extraneous cognitive load [42]. When teaching through virtual worlds, it is essential to consider the extraneous cognitive load as, if the student does not have the ability to function easily in digital systems, their learning may be negatively affected [41].

### SL From a Higher Education Perspective

#### Overview

Virtual worlds have a remarkable potential for effective teaching and learning [43,44]. These experiential environments bring the possibility to create immersive, realistic, and engaging web-based events that can provide high-quality medical education to health-related users in remote locations [45-47].

SL is considered the most popular virtual world among educators and the most used in higher education [25,48-51], particularly in the education of medical professionals [43,52]. It can be seen from the literature that most of the studies on the application of virtual worlds in higher education are based on SL [53].

#### Advantages and Potential for Learning

SL is free to use for participants (students and teachers), and it may not be complex to manage for young users such as medical students. The components of the SL experience can facilitate educational innovations through the following [25]:

- *Expanded or rich interactions*: referring to social interaction between individuals and communities as well as between humans and virtual objects
- *Visualization and contextualization*: the production and reproduction of content that is inaccessible, too expensive, imaginary, futuristic, or impossible to see for the human eye
- *Exposure to authentic content*: scientific, technical, and cultural
- *Identity play*: both individual and collective
- *Immersion in a 3D environment*: the sensation of presence through virtual embodiment in the form of an avatar and extensive modes of communication can impact the affective, empathic, and motivational aspects of the experience
- *Simulation*: reproduction of contexts that may be too expensive to reproduce in real life, with the advantage that some physical limitations can be overcome
- *Community presence*: promoting a sense of belonging and purpose that unites groups, subcultures, and geography
- *Content production*: opportunities for the creation and ownership of the learning environment and the objects within it

#### Barriers and Limitations

Although there is abundant evidence that educational activities can be carried out in scenarios specifically designed in SL, it must be recognized that there are certain barriers that may limit its use. One problem with using SL for education may lie in the technical capabilities of some users’ computers and internet connections, because the SL viewer has significant requirements to run adequately [54]. There is a necessary learning curve for users; few people can simply “jump” into SL for the first time effortlessly [17]. This may cause rejection in some participants. In contrast, SL is considered old technology by some young users; for example, it still does not have an official native mobile app. However, there is an imminent release of an iOS or Android app for SL running on Unity, bringing the venerable virtual world “in top form” into the modern era [2].

There are other cultural, social, time investment, or economic barriers that must be considered. For example, as a result of the global reach of the virtual world, cultural and linguistic differences must be considered [17]. SL can be an isolating experience for the user as it is not always easy to participate and integrate into new communities in a place outside the user’s “safety zone.” Nonverbal cues with avatars are almost impossible to obtain (aside from user-generated emoticons). Time can be a considerable barrier as creating content and designing, validating, and executing educational activities requires time to address issues such as intellectual property rights, object permissions, and accessibility [25]. Although SL is free for learners, with basic accounts, the overheads of designing, implementing, practicing, and maintaining virtual sites in SL often require educators to develop multiple skills.

#### Universities in SL

**Figure 2:**
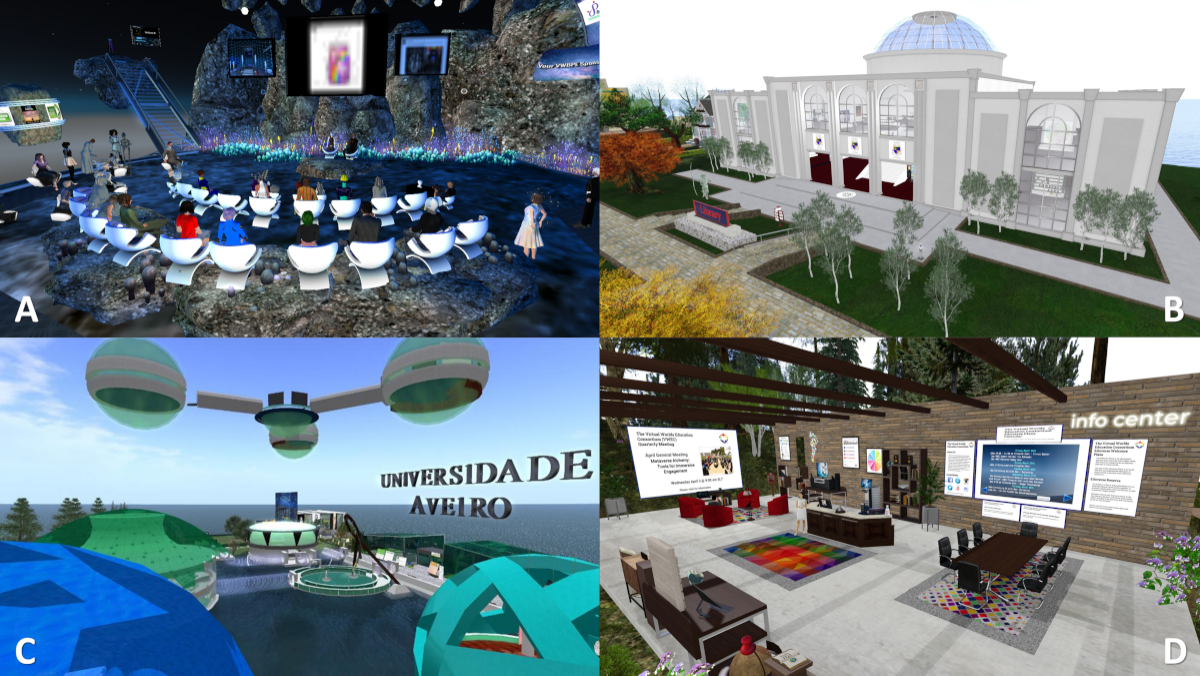
Screenshots of university organizations in Second Life. (A) A lecture at the 2020 Virtual World Best Practices in Education meeting. (B) The Rockcliffe University Consortium library building. (C) A view of the island of the University of Aveiro. (D) The meeting point of the Eduverse campus of the Virtual Worlds Education Consortium.

The pedagogical possibilities of virtual worlds have motivated educators so that colleges and universities worldwide have taken the initiative to develop virtual campuses in SL. The early years of SL were very active in terms of education-related activities, with enthusiastic development. As of early 2007, a total of 98 colleges and universities worldwide had a presence in SL, a number that increased to 250 in 2009 [17]. In 2010, SL listed 78 universities as members [32], and >150 academic organizations were included in the SL Education Directory by 2013 [55]. At the time of writing this manuscript, a search inside SL using the term “university” returned 102 entries for venues or destination guides, giving a clear idea about the increase in the use of this environment for teaching and learning projects. Among them, it is worth highlighting the Rockcliffe University Consortium (Figure 2), which was started in 2006 by Phelan Corrimal with the goal of providing experiential educational opportunities in SL. In 2009, Rockcliffe had a virtual campus, widespread interaction with the nonprofit SL community, participation from 40 to 50 real-world universities, and a peer-reviewed journal (*Journal of Virtual Studies*). Rockcliffe is the organizer of the Virtual World Best Practices in Education Conference in SL (Figure 2), with 17 annual editions uninterruptedly [56], having reached 4000 attendees. Rockcliffe continues to actively pursue its mission of providing an environment for educational innovation, including affordability for students and teachers [17].

The Virtual Worlds Education Consortium launched a 7-region virtual educational campus called Eduverse in 2022 (Figure 2) comprising a group of virtual places focused on education in SL [57]. The other regions that are part of this project include The Science Circle, Virtech, the Community Virtual Library’s Cookie region, the Mayo Clinic, Whole Brain Health’s Inspiration Island region, and the University of New Mexico at Rockcliffe’s Rockcliffe Village region. Since then, the consortium has held several weekly meetings nonstop on the shared campus with the goal of helping groups already in SL increase collaboration and resource sharing. The Virtual Worlds Education Consortium’s purpose is to bring educators in virtual worlds together both to share what has been successful and tackle obstacles across different platforms and communities [58].

#### Educational Possibilities

The SL platform allows one to reproduce typical classroom learning activities such as courses, seminars, and workshops [33]. In the authors’ opinion, SL offers certain advantages over other 2D web-based learning tools, as synchronous learning experiences can be organized with very diverse formats in varied and imaginative environments. Furthermore, it represents an alternative to these 2D teaching resources, which, when used very frequently during the COVID-19 pandemic, produced a certain saturation among users [59].

SL allows for interesting simulation and gamification experiences that are impossible (or very expensive) to do in real life [25,33], with the added value of generating more dynamic contact, which is even more fun for Generation Z. There are various educational formats that can be carried out in the SL environment, adjusting to various learning areas in science, technology, and the humanities. These could be summarized as follows [25]: (1) self-paced tutorials, (2) displays and exhibits, (3) immersive exhibits, (4) role-plays and simulations, (5) data visualizations and simulations, (6) historical recreations and reenactments, (7) living and immersive archaeology, (8) machinima construction, (9) treasure hunts and quests, (9) language and cultural immersion, and (10) creative writing.

## Medical Education Within SL

### Learning Activities in Medical Education

#### Overview

A narrative review of the existing literature was carried out, searching for information in the PubMed and Google Scholar databases using the terms “Medical Education” AND “Second Life.” In addition, the point of view of our teaching team was considered after uninterrupted practical experience in undergraduate and postgraduate medical education in SL during the last 13 years with >4000 learners and >10 publications on the subject.

There are a wide range of health-related activities on SL, most of them dedicated to patient education, raising awareness of health issues, patients with specific diseases, marketing and promotion of health services, or health research. There are also academic sites offering training by means of classrooms, discussions, lectures, simulation, and patient interaction [60,61]. This review focused especially on medical education experiences during undergraduate, residency, and continuing medical education (CME). Multimedia Appendix 1 [33,45,46,62-88] shows a descriptive summary of the articles found. Studies related to the training of other professions such as nursing, psychology, and veterinary medicine were outside the focus of this review.

Different learning methodologies have been adopted tailored to varied groups of health learners. Some of them focus on developing immersive and realistic virtual patients or simulation scenarios within SL, training paramedic students in emergencies [89], the management of specific clinical situations by nurses [90], or the practice of communication and assessment skills by mental health nursing students [91]. Others focus on holding training workshops and clinical sessions [46] or interactive seminars in SL for postgraduate primary care physicians [45] or using the SL virtual environment for mock oral examinations for emergency medicine residents [62]. Academic experiences tailored to medical students performed in SL include creating a virtual laboratory for web-based anatomical education [63] or evaluating the use of team-based learning (TBL) of anatomy [64]. SL is also useful for conducting web-based radiology training activities with remote access in an attractive setting, especially for current generations of medical students and residents [33]. Typical classroom activities such as courses, seminars, and lectures can be transferred to SL, taking advantage of the feeling of “being there,” but active learning methods, which are more attractive to the student, can also be included, such as gamification, problem-based learning (PBL), or medical simulation. The development and characteristics of some of these activities in SL are explained in the following sections.

#### Courses, Seminars, and Conferences

**Figure 3:**
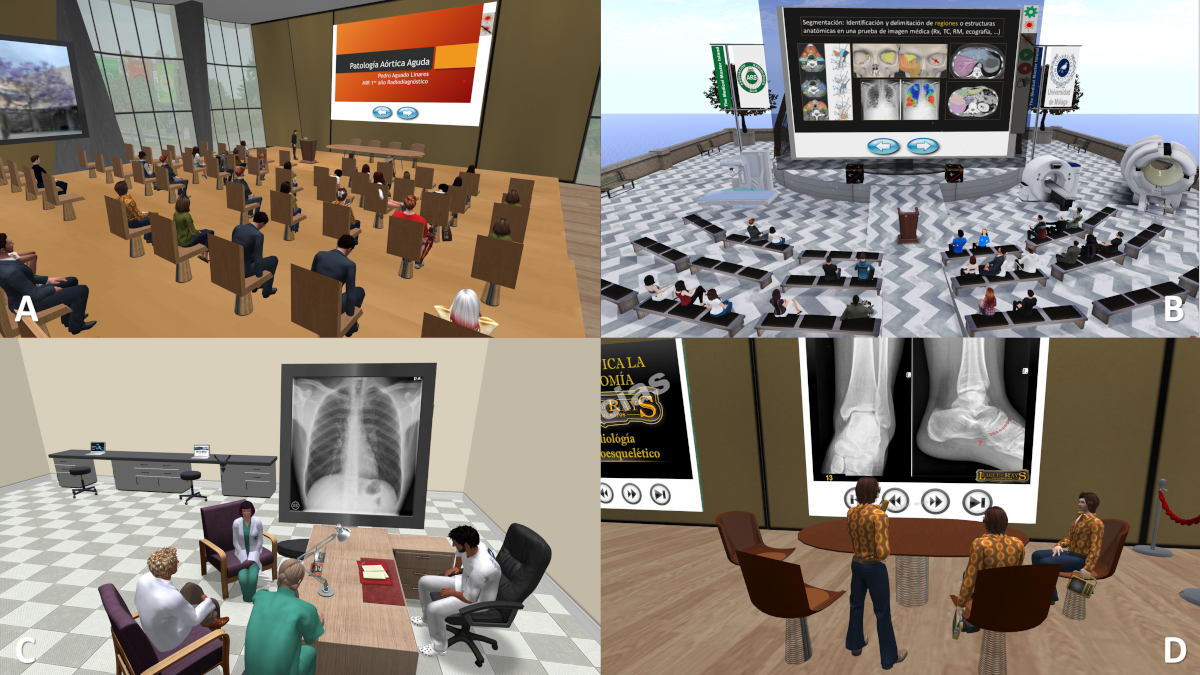
Screenshots of various scenes during medical education activities in Second Life. (A and B) Lectures with groups of approximately 30 avatars in a virtual classroom and an outdoor setting. (C and D) Scenes of student group meetings to discuss and prepare radiological cases.

SL has remarkable potential for effective teaching and learning as its structure allows for synchronous web-based meetings between teachers and students in the form of lectures, courses, or other typical classroom work formats [33]. These activities can be carried out in very varied scenarios, recreating a classroom, an auditorium, an open stage next to the sea, a floating platform in the sky, and infinite possibilities with the only limit of imagination and time to create these scenarios (Figure 3). Lorenzo-Álvarez et al [65] conducted a randomized study with medical students comparing an x-ray interpretation workshop conducted in SL and in real life. The results of the pre- and postexposure knowledge tests demonstrated that learning from a synchronous educational event in SL did not show differences from learning in the real world if the same content and script are maintained.

In addition, the persistence of the system (open 24/7) allows for the inclusion of asynchronous activities to be solved during a course at the students’ own pace [33]. The combination of synchronous sessions with asynchronous tasks is very interesting and useful for organizing web-based courses in SL. This type of organization of courses in SL has been highly valued by medical students with average scores of 8.9 to 9.3 out of 10 points [66,67] and by postgraduate physicians with average scores of 8.7 to 9.1 out of 10 points, who also perceive the usefulness of this platform in the training of residents and CME as it adapts to their work schedule and minimizes travel costs [67,68].

#### The Teacher’s Perspective

Remote access allows for inviting speakers or professors from different institutions, opening up new interesting and enriching avenues for educational contact with students and between professors from different universities, with the advantages of reducing costs and travel times. From the perspective of 9 medical teachers who were invited to give a seminar in SL for the first time, it represents a training experience for trainers, which allowed them to learn about a new web-based infrastructure with interesting synchronous teaching capabilities (time, accessibility, ubiquity, synchrony, facilities, sound, and 3-dimensionality) [69]. The perceptions of 14 medical teachers after a 3-hour workshop given in SL explaining to them the platform and its educational possibilities “from within” were very positive [70]. They all considered it interesting and useful for teaching, and 71% agreed to carry out a teaching activity in SL. It is important to develop future actions in SL aimed at the training of trainers to [70] (1) promote collaborative work between teachers, (2) familiarize teachers with the educational possibilities of virtual worlds and the basic applicable technology, (3) share teaching experiences carried out in SL to achieve new interdisciplinary collaborative projects, (4) use SL as a means of virtual meeting and discussion, and (5) promote the development of multidisciplinary and interuniversity collaborative educational projects.

#### PBL Method

PBL, developed by Barrows [92] at McMaster University in the 1960s, is a student-centered learning methodology in which students are faced with a problem situation that they must solve themselves by applying previous knowledge and incorporating new identified knowledge [93]. The teacher stops being the center of the educational experience and becomes a guide who facilitates and directs learning. In medical education, this methodology contributes to acquiring skills such as real-world clinical reasoning, problem-solving, and critical decision-making [94]. In medicine, these “problems” are usually generated in the form of clinical cases, creating a variant of PBL known as case-based learning [95].

SL offers good support for posing open-ended problems to students in a PBL context [20]. PBL experiences have been carried out in SL with paramedic students [96] and medical students [71,72]. Beaumont et al [96] developed PBL scenarios about medical emergencies in SL. Results with focus groups of 10 students showed that SL can provide a rich and engaging environment based on the authenticity of the scenarios. As disadvantages, they pointed out a certain lack of detailed realism, the lack of face-to-face and hands-on contact in the experience, and some access problems. Rampling et al [71] designed a virtual patient with psychosis in SL for PBL in psychiatry. Participation was low (only 24 students out of 150 were invited), and feedback was predominantly negative, with 53 critical responses against 32 positive ones. Students expressed that the scenario was cumbersome, did not imitate real life, and had little educational value. The authors understood that the problem lay in the use of scenarios within SL based predominantly on verbal communication. Jivram et al [72] studied the use of SL to enhance PBL in medical education comparing it to interactive web-based methods. Although a minority of the 244 participating students stated that the SL experience seemed more realistic, most preferred web-based methods due to their simplicity and effectiveness. The authors indicated that this was partly due to the temporal proximity of the exams and the additional effort required to learn the SL interface. These experiences show the way toward the development of PBL sessions in 3D virtual worlds in medicine. The technological evolution of SL in recent years can improve the authenticity of the scenarios [2], although there are access and usability issues that could be minimized by scheduling the experiences appropriately in the academic calendar and by training in the use of the platform and verbal communication.

#### Communication Skills

Communication features in SL simulate real-world communication. For example, voice volume is louder or weaker as the avatar moves closer or further away from the speaker and is perceived from the right or left depending on where the speaker is located [59]. This brings great realism to the immersive experience, and therefore, SL is an ideal tool for developing oral communication skills. Mitchell et al [73] conducted a pilot study in which a motivational interviewing program in SL was designed and tested to counsel patients about colorectal cancer screening, the results of which suggest that virtual worlds offer potential to enhance patient-centered communication skill training. Other experiences have shown that SL is an excellent tool for training in transversal communication skills such as public oral presentation of a topic, interaction with other users in real time, and the establishment of links and social relationships between attendees both in undergraduate [97] and postgraduate [74] students.

Meetings in SL allow for feedback from the audience, which, along with expert observation, are elements that help improve public presentation and communication skills [98]. van Ginkel et al [99] conducted a study with 36 first-year college students who completed a required oral presentation course in a virtual environment created using Unity. We agree with these authors that this type of virtual experience imitating real life helps further develop oral presentation skills, but it is essential that attendees learn to correctly use the basic audio communication controls (microphone and headphones) and avatar vision to properly follow the development of a session [74,97].

The presenter’s voice is literally the instrument of connection with the attendees. When they give a presentation, how the audience feels about their voice is integral to how they perceive the presentation [98]. The quality of audio in SL allows for interaction, evaluation, and improvement of oral expression. Although, on the one hand, there is the disadvantage of not perceiving gestural communication, on the other hand, the filter provided by the interface and the avatar in SL makes beginners feel less embarrassed when speaking in public [97] and express themselves more openly and honestly [17].

#### Assessment

Assessment is an essential element in the teaching-learning process. SL allows teachers to carry out various exam formats in-world efficiently. Note cards are a document that records the user who created them and what time they were delivered to the teacher’s avatar. Using note cards, deferred evaluations can be carried out so that the student can solve the assigned questions in writing in an allotted time [66-68] or multiple-choice tests in real time in the virtual classroom, with a short time assigned to each question [65,68].

The oral examination is a traditional method for assessing medical knowledge, clinical reasoning, and interpersonal skills. Schwaab et al [62] conducted an interesting study in which 27 emergency medicine residents participated in a virtual oral exam in SL. All examinees felt comfortable communicating with the examiner; most thought that the SL encounter was realistic (92.6%); and all felt that the virtual exam was fair, objective, and conducted efficiently. Most (66.6%) preferred to take oral exams using SL rather than the traditional format and expressed interest in using SL for other educational experiences (92.6%). McGrath et al [75] carried out a similar experience with 35 residents also from emergency medicine randomly assigned to SL (n=18) or the real world (n=17). Examinees in both groups scored without significant differences and thought their evaluation was realistic, fair, objective, and efficient. However, examinees in the SL group reported a preference for the virtual format and felt that it was less intimidating.

#### Simulation

Simulation is increasingly used in health care areas, especially in undergraduate education as a starting point for training medical professionals. Simulation-based learning experiences are useful for integrating theoretical knowledge with practice and the necessary practical skills [100-102]. Virtual simulation experiences have been developed in SL and other virtual worlds to train various skills, such as taking anamnesis with virtual patients [76], solving clinical situations in a pulmonology ward [77], conducting cardiopulmonary resuscitation [103], or developing communication skills with patients [104]. Danforth et al [76] used SL as an immersive environment to develop a standardized virtual patient system and an artificial intelligence (AI) markup language as a dialogue engine. This first system was useful to demonstrate the viability of the approach, but the virtual environment and dialogue management at the time (2009) were not ideal. To manage conversations, they used >200,000 rules due to inefficiencies in markup language and AI syntax. For this reason, they redesigned the application using Unity 3D to create the virtual environments and implemented ChatScript to manage the dialogue [105]. Students were able to accurately develop an appropriate differential diagnosis based on information provided by the simulated patient during the encounter, with the accuracy of simulated patient responses ranging from 79% to 86% depending on case complexity, type of history obtained, and student skill.

Toro-Troconis [77] at Imperial College London created a game-based SL simulation for medical students in which they could interact with virtual pulmonology patients to develop their skills and confidence. One group (n=23) had access to the virtual patients in SL, and another (n=19) used an interactive electronic module. Similar attitudes were observed in both groups, with women showing a more positive attitude toward the perceived usefulness component of virtual patients in SL. The repetitive linear presentation of cases was not sufficiently motivating, so the use of more challenging branching learning experiences for the presentation of virtual patients is recommended [77].

Virtual simulated patients have the potential to reduce costs, faculty time, and resources required to help students develop their communication skills in safe, nonthreatening environments before contact with real patients. They also allow for standardization of interactions among students. Current efforts should focus on adding fluency to communication with virtual patients and feedback and assessment capabilities so that students can receive immediate feedback on the quality of their encounters with virtual patients [105].

#### Objective Structured Clinical Examination

The objective structured clinical examination (OSCE) is an examination format developed in different physical spaces (called clinical stations) in which clinical scenarios and situations are reproduced to evaluate the student’s clinical skills in a standardized, reliable, and objective manner [106,107]. The restrictions on contact and physical presence during the COVID-19 pandemic favored 2D virtual OSCEs, with web-based access as a solution to these restrictions [108-110] proving to be enjoyable, interactive, and easy for students, as well as cost-effective [111].

SL is a useful digital platform for carrying out avatar-mediated OSCEs, both summative and formative, as it makes it possible to design and recreate various scenarios to train decision-making in clinical cases [72]. This has been verified in domestic accident scenarios in geriatric medicine, an experience in which 6 of the 8 geriatric medicine fellows rated the simulation as “excellent” [78], or in office or hospital settings, where 12 urology residents found the method feasible, acceptable, and applicable to assess communication skills [79].

**Figure 4:**
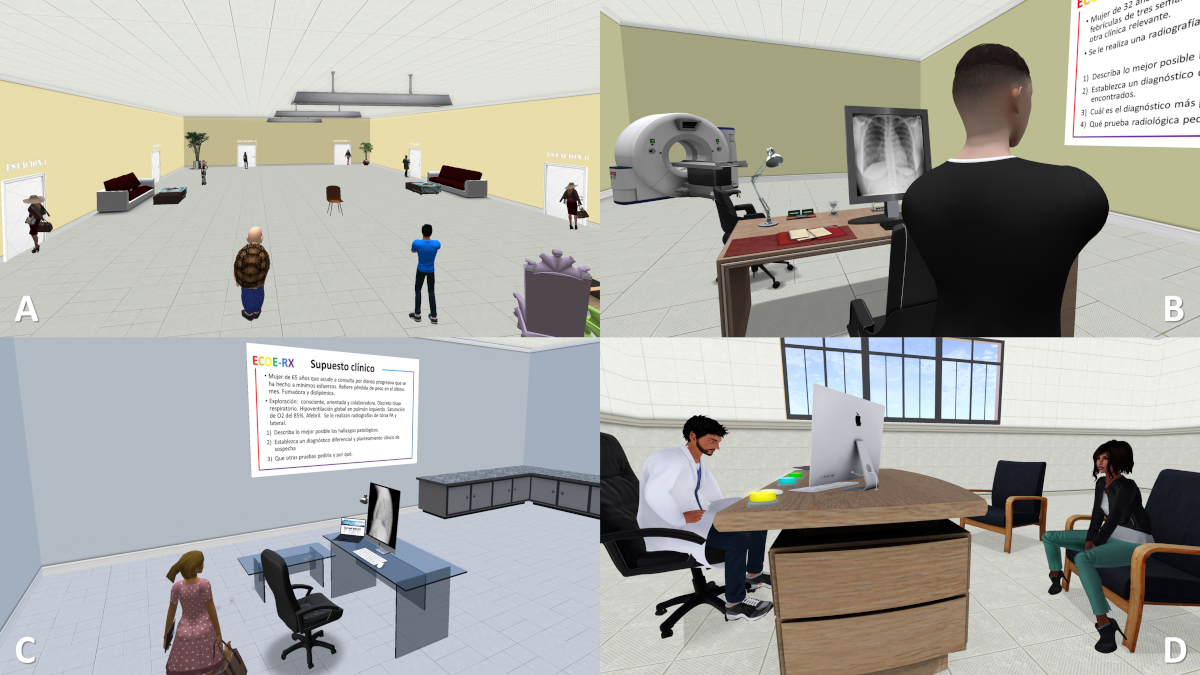
Screenshots of various scenes during a virtual objective structured clinical examination (OSCE) in Second Life. (A) Distribution of an OSCE room with students in front of the door of each station before starting. (B) Scene of a student at a radiology station. (C) Scene of a student reading the clinical scenario presented on a poster on the wall. (D) Scene in which the student interacts with a simulated patient represented by another avatar.

Recently, a teaching experience in SL on emergency radiology was carried out with 180 sixth-year medical students organized into groups of 3 to 4 who had to solve emergency radiology shown in virtual OSCE stations. The students assessed the OSCE environment, the cases, the organization, and the training usefulness with average scores of 8.5 to 8.9 points out of 10 [80]. Currently, the authors are working on the development of a virtual radiology OSCE in which students must complete 6 radiology stations (Figure 4) answering questions about radiological interpretation, clinical judgment, and patient management having 9 minutes per station, replicating the organization of the face-to-face OSCE at the end of the degree. It would be interesting to develop and implement radiological OSCEs in virtual environments such as SL at the undergraduate, residency, and CME levels as it would allow for the optimization of resources and the performance of multicenter studies [112].

#### Gamification

Gamification applies elements of playful design to nonplayful contexts such as learning [113,114]. Gamification can be carried out in the classroom but also using web-based resources that facilitate remote access and user management, including the metaverse [115]. The SL platform offers experiences that are a game in themselves. Users enter an imaginary world in which they move and interact represented by avatars whose appearance they can modify at will. Gamification in SL has interesting applications in medical education. Toro-Troconis [77] developed an interesting study on gamification with virtual patients in SL, among whose conclusions stand out the following: the importance of following a pedagogical framework in the design of game-based learning activities, the effectiveness of giving feedback to participants about rewards and their progress in the game, and the recommendation of previous exposure of students to the SL region where the activity will take place so that they get used to the environment and can concentrate on learning.

In 2015, a competitive web-based game in SL called League of Rays (LOR) [81] was designed to provide medical students with a gamified complement to the formal teaching of radiological anatomy and radiological semiology. The game, which lasts for 6 weeks, takes place on an island where, every week, 5-m panels with educational content appear, which are replaced by multiple-choice tests and other tasks to score points. In the first edition, voluntary participants scored better than nonparticipants in a postexposure test performed 1 month after the game (mean scores 59.0, SD 13.5 vs 45.3, SD 11.5; *P*<.001), indicating effective learning with the game [81]. In addition, both participants and nonparticipants provided valuable positive perceptions of the game and its benefit to their education. The following 2 years, participation was mandatory [82], demonstrating slightly less positive perception of and lower adherence to the game than when participation was voluntary. Subsequently, the rules were modified to voluntarily participate in teams of 4 students, encouraging teamwork and collaboration [83], and finally, 2 editions of interuniversity competition were held [84] in which the reproducibility of the experience was demonstrated, ruling out proximity biases and promoting collaborative action between teachers. Participants found team gaming useful for maintaining virtual relationships and identified this activity as a playful learning and social interaction experience during the COVID-19 pandemic [84]. This experience, maintained for 8 consecutive years and completed by 1032 students, demonstrates the usability of SL for learning games and the possibilities for collaborative work between students during gamification. In May 2024, a game similar to LOR designed for radiology residents called “Resident Debil” was developed in SL, the results of which are pending evaluation.

### Health-Related Locations in SL

Relevant institutions have explored how to promote initiatives to improve health care by creating their own locations in SL. Some of these locations are not currently active. For example, the “Isla de la Salud” hosted interesting educational experiences with family physicians [46]; it even hosted, in parallel to real life, national congresses of the Spanish Society of Family and Community Medicine between 2008 and 2010, but unfortunately, it no longer exists today. At Ohio University’s SL location, a nutrition game was developed in which people could learn about the impact that fast food has on health [43]. The University of London created a virtual hospital in SL that included operating rooms where, for example, some posters showed the students the steps to prepare for surgery (such as dressing, wearing a mask, and disinfecting after the virtual procedure). Other parts of the hospital, such as a polyclinic or virtual pharmacy, were built in the same way to provide information to the students in an immersive environment [116].

**Figure 5:**
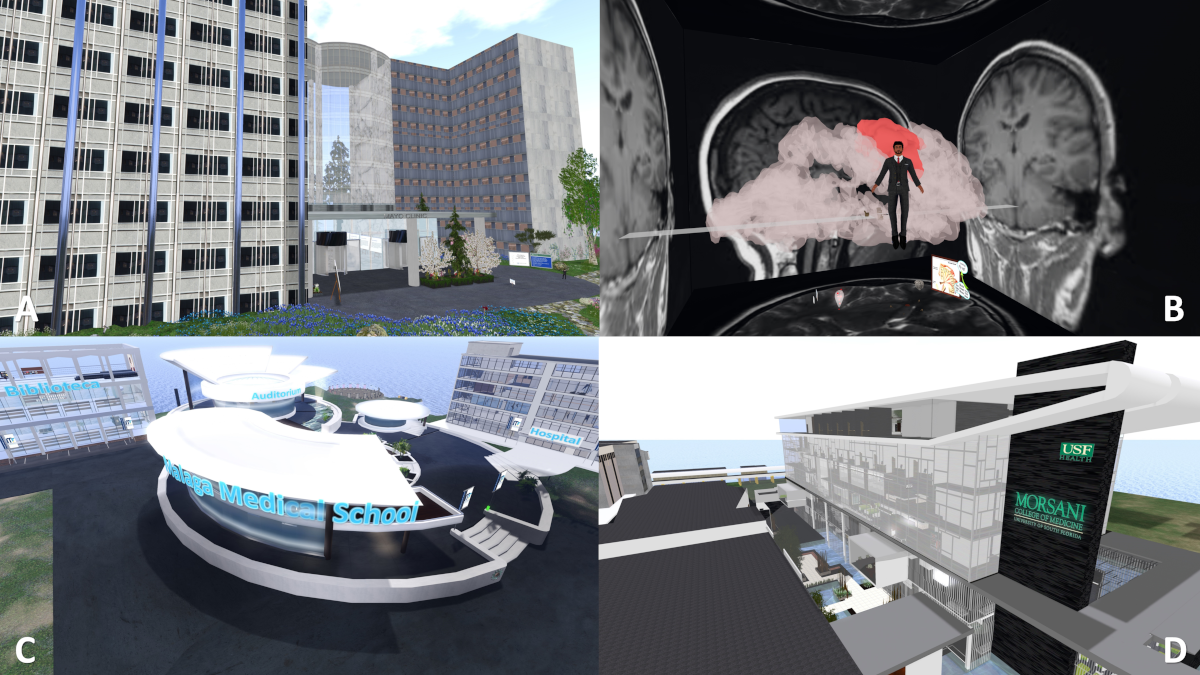
Screenshots of several health-related virtual locations that are currently active in Second Life. (A) Mayo Clinic hospital. (B) Brodmann’s Brain at Inspiration Bay, representing functional areas of the human brain. (C) Malaga Medical School island. (D) The University of South Florida College of Medicine.

There are other health-related virtual places that are currently active (Figure 5). The Mayo Clinic island in SL [117] includes conference facilities and a bookstore. The nonprofit medical practice regularly hosts talks and events on illnesses and diseases for interested users. The University of South Florida [118] College of Medicine headquarters includes information about the various schools and departments, including nursing, public health, and continuing education. Brodmann’s Brain at Inspiration Bay [119], developed by a team of virtual creators and neuroscientists, allows the visitor to walk through a gigantic interactive construction that shows functional areas of the human brain, which are activated (lighted up) based on various functions such as dancing or loving. The Málaga Medical School island [120] was created in 2021 to host permanent medical education content, synchronous educational events, and multidisciplinary OSCEs for undergraduate and graduate students.

### Educational Experiences in Different Medical Disciplines

#### Anatomy

Interesting experiences have been carried out for teaching anatomy in SL. Morales-Vadillo et al [121] used 3D scale models of oral anatomy created in SL that dental students were able to use without limitation for 4 weeks. The experimental group (n=23) scored significantly higher on questions about conceptual understanding and spatial interpretation than the control group (n=37) that used traditional teaching methods. Other authors have also created anatomical models so that students could evaluate them from the view of their avatar. Danforth [85] made a virtual testis model not only to explore the anatomy but also to examine the relationships between anatomy and physiology to describe the sperm production function. Richardson-Hatcher et al [86] built a 3D replica of the pterygopalatine fossa in SL where the students could virtually experience the organization of this anatomical area, maneuvering in and out of its boundaries and following the course of the nerves through the fossa. The study data suggest that students view this new technology as a valid method for studying anatomical arrangements by improving their understanding.

Gazave and Hatcher [64] implemented virtual TBL in a web-based anatomy course held in SL with 39 medical students. They demonstrated that virtual TBL was engaging for most students (average engagement score of 7.8 out of 10, with 89.2% of students reporting a score of ≥6). The authors concluded that virtual TBL sessions are worth implementing in other web-based courses. Richardson et al [63] built a multipurpose virtual anatomy laboratory in SL with a series of stations with (1) atlas or cadaver images, (2) a tour of cross-sectional anatomy, (3) cadaveric anatomy video tutorials, and (4) group quizzes. Students could obtain note cards with information and questions for each station by clicking on the station header. The authors stated that, while the virtual laboratory is not a substitute for cadaver-based anatomy experiences, SL allows students to participate to a greater extent than typical lecture transmissions via web-based learning.

#### Primary Care Medicine

Wiecha et al [45] designed a pilot postgraduate medical education program in the virtual world in which 14 primary care physicians participated. It was a 1-hour interactive session in SL on the topic of type 2 diabetes. Lorenzo-Álvarez et al [67] conducted a 3-week course for 14 family physicians on plain radiograph interpretation. Both studies reached similar results, with all participants agreeing that the SL experience was an effective CME method and that they were willing to participate in further educational events in SL. Melús-Palazón et al [46] carried out a series of accredited virtual clinical sessions in SL in which 76 primary care professionals from 9 health centers participated. They concluded that SL is a tool that allows for the design of educational activities that involve several health centers in different geographical locations, thus eliminating the need for travel and making more effective use of educational resources.

#### Psychiatry

There have been some experiences of medical education in psychiatry conducted in SL. In 2006, Yellowlees and Cook [122] built a visual and auditory hallucination recreation experience in SL in which 76% of 579 SL users reported a better understanding of visual and auditory hallucinations. The authors concluded that simulations of the perceptual phenomena of psychiatric illnesses were feasible with the computer technology existing at that time. Rampling et al [71] used SL to create a simulated patient with psychosis for clinical training of medical students. A total of 24 participating students gave predominantly negative evaluations of the experience, and the authors did not consider it advisable to use scenarios predominantly based on verbal communication with the patient within SL, proposing to explore other interactive teaching methods in psychiatry, such as PBL.

#### Radiology

The use of e-learning and web-based resources to deliver radiology teaching to medical students represents an exciting alternative and an effective method to improve radiological knowledge and skills [123,124]. Medical images can be displayed in SL with sufficient quality to interpret the findings they present, providing adequate support for carrying out educational activities in radiology [33]. In 2011, a virtual space called “Medical Master’s Island” was created [125], and various radiology teaching activities have been carried out uninterruptedly since then. More than 4000 learners, undergraduate and graduate, participated in these activities until May 2024. The most recent experiences are still pending publication.

In 2011, a pilot study was conducted with 46 medical students that demonstrated the feasibility of teaching undergraduate radiology in SL and the great acceptance by students [66]. In 2012 and 2013, several basic radiology courses were held in which 48 third-year students participated [67]. Both experiences demonstrated that students highly valued SL as an educational platform, recognizing the effort made by teachers to provide them with this opportunity, with average scores of 9.6 to 9.8 out of 10 points. In 2014, a randomized study was conducted with 156 third-year students to answer the following question: is learning in SL the same as learning in the real classroom [65]? An abdominal radiology seminar was given, and no significant differences were found between both groups in the pre- and postexposure tests, concluding that radiology education in SL encourages participatory learning and results in an acquisition of interpretive skills similar to that of a traditional face-to-face class.

As explained in the *Gamification* section, 8 consecutive annual editions of the multiuser game LOR, designed for learning radiological anatomy and semiology, were carried out between 2015 and 2022. It started as an experience in a single university [81-83], continuing as an interuniversity competition [84] in which, for 3 years, 129 teams of 4 students from 20 different universities participated. These experiences show the applicability of remote access to asynchronous gamification thanks to the persistence of the platform. Recently, in 2021 and 2023, “classroom-typical” synchronous gamification experiences have been carried out in SL, playing games such as Trivial Pursuit and The Alphabet to learn radiology [87].

Since 2011, other radiology training activities have been carried out, such as x-ray interpretation courses for 96 family medicine residents and 67 radiology residents from all over Spain [68], PBL experiences in radiology with medical students, and other activities based on training communication and public speaking skills on radiology topics with undergraduate and graduate students. Currently, the activity of this group from the University of Málaga is focused on medical simulation and carrying out virtual OSCEs in SL. In addition, the aim is to establish institutional alliances to promote the culture of virtual worlds between teachers and students.

#### Impact of the COVID-19 Pandemic on Medical Education in SL

The COVID-19 pandemic accelerated the application of new technologies in learning [126], caused stress in medical students [127], and reduced physician-student contact [128]. Various 2D platforms, which enable synchronous educational experiences between teachers and students, have experienced great technological development since the COVID-19 pandemic [129-133]. One might ask, if these 2D remote connection methods already exist, why are 3D environments needed? On the one hand, 3D environments such as SL have some advantages over synchronous 2D communication platforms [25,134] as they (1) are a 24/7 persistent environment; (2) allow for immediate, real-time interactions between users and objects in the 3D environment; (3) induce a strong feeling of presence (sense of “being there”) in users; (4) promote social awareness, or the ability to sense the presence and location of participants in a learning environment, reinforcing the perception of “who is there” and “what is happening”; and (5) generate a greater sense of belonging to a community. On the other hand, there is a phenomenon of fatigue from 2D videoconferencing triggered by the intensive use of videoconferencing since the COVID-19 pandemic, so 3D environments appear as an alternative for a change of scenery [59].

The need to continue the teaching-learning process during the pandemic and the limitations of 2D virtual modalities, in which it is difficult to carry out experiential learning, led to the development of learning scenarios through the metaverse and virtual worlds [135]. Thanks to the availability of precreated SL resources when the lockdown was declared, the planned radiology seminars for 157 medical students could be quickly transferred to SL [88]. More than 90% of them felt more engaged in teaching thanks to the SL seminars and stated that they found the virtual platform an attractive and fun environment and that, if they were to return to a lockdown situation, they would like to conduct similar learning activities [88]. Furthermore, participants in the 2020 interuniversity LOR competition found the team game useful for maintaining virtual relationships and identified this activity as a playful learning experience and social interaction during the COVID-19 pandemic [84].

#### Limitations and Drawbacks

Although, in general, both students and teachers involved have valued the use of SL for teaching medicine very positively, there are some limitations and drawbacks that should be pointed out. First, technical limitations due to the low capacity of the central processing unit, graphic card, or internet connection can prevent access to SL or cause defects in the representation of the virtual world [88]. These types of incidents have been described by 9% to 11% of participants [65,66,81] and are not always critical. Second, when the audio quality (voice input or output) of a user is limited, it makes it difficult to correctly develop synchronous activities with voice support, something crucial when it happens to the teacher. Therefore, it is advisable to train and check the equipment to be used beforehand [88]. Third, it is sometimes difficult to view the same presentation, especially when there are many interactions on the display screen. This can be minimized with adequate previous training explaining the rules of behavior during the sessions [88]. Fourth, the limit of attending avatars on an island is 100, but to avoid access and interaction problems, it is recommended not to exceed 45 to 50 avatars synchronously in the same place [88]. This limit prevents synchronous activities with large groups in SL.

## The Future

The metaverse is alive, and so is SL. Constant technical improvements are sought. Virtual world data are no longer housed in large server rooms but are hosted in the Amazon cloud. Linden Lab is a more decentralized company based on the remote work of its employees. The company is slowly rolling out its “Puppetry Project,” a new feature that allows the user to capture their real movements and facial expressions via a webcam and other motion capture devices and then display them on their avatar in real time [2].

The contributions of the community of creators also offer technological changes for the immediate future. For example, SL viewers with more capabilities continue to be developed. The SL viewer and the third-party Firestorm viewer are usually updated to a new version several times a year. Crystal Frost, a new external SL viewer running on Unity designed by Berry Bunny, an SL community creator, is in its beta phase for Windows but will also be available on Linux and Mac and on mobile devices [136].

Other 3D collaborative tools are currently being developed, such as Frame VR [137], in which users share their virtual space to work together. The future integration of technologies such as VR and AR in platforms such as SL and Frame VR opens interesting lines of research in education. As VR technology allows users to fully immerse themselves in a computer-generated world and AR overlays digital information onto the real world, their combination in virtual worlds could lead to a new era of virtual presence in which learners could interact with the virtual environment and other avatars in more natural and intuitive ways [138]. As these technologies continue to evolve, they are likely to become more integrated into our daily lives toward a fusion model, blurring the lines between physical and digital realities [139]. However, the successful integration of these technologies requires addressing challenges related to accessibility, technical requirements, and the development of effective pedagogical strategies [138].

Generative AI can also bring exciting possibilities to medical education in SL, for example, AI-powered chatbots. This would allow for more immersive human-computer interface loops, from conversations with patients or virtual therapists to interactions of very diverse types. Since there is user input, AI could be trained to produce highly customizable responses or generate unique stories per use [2].

## Conclusions

In the 25 years since its conception, SL has proved to be a virtual platform that connects with the concept of the metaverse, an open system with global access and interconnected where all humans can access to socialize or share products for free or using a virtual currency. SL continues to be successful as a virtual world model that connects with the concept of an equitable, diverse, and creative metaverse. Its creativity tools are open and powerful, and its user community is diverse and made up of members free to create endless values and possibilities.

These 25 years have made SL a platform where different generations coexist. SL remains active and technologically improved since its creation. Future improvements are also expected, such as the creation of viewers for iOS and Android that will allow SL to be used on mobile devices or the incorporation of generative AI, which will provide more enriching and nonrepetitive experiences.

From an educational point of view, SL has the advantages of being persistent 24/7 and creating in the student the important feeling of “being there” and of copresence. This, together with the reproduction of the 3D world, real-time interaction, and the quality of voice communication, makes immersive experiences unique, generating engagement and a fluid interrelation of students with each other and with their teachers. Various groups of researchers in medical education have developed experiences during these years, which have shown that courses, seminars, workshops and conferences, PBL experiences, evaluations, teamwork, gamification, medical simulation, and virtual OSCEs can be successfully carried out. Acceptance by students and teachers is generally positive, recognizing its usefulness for undergraduate medical education and CME. However, there are few studies evaluating the educational impact of SL activities. Therefore, it is necessary to continue carrying out educational experiences, outlining the organization, objectives, and content and measuring the real educational impact to make SL an educational tool for more universal use.

## Appendix

Multimedia Appendix 1 Articles related to medical education experiences in Second Life during undergraduate, residency, and continuing medical education.

## Abbreviations

AI: artificial intelligence
AR: augmented reality
CME: continuing medical education
LOR: League of Rays
MMORPG: massively multiplayer online role-playing game
OSCE: objective structured clinical examination
PBL: problem-based learning
SL: Second Life
TBL: team-based learning
VR: virtual reality
XR: extended reality
