# Supplementary material for: The Second Life Metaverse and Its Usefulness in Medical Education After a Quarter of a Century
Source: J Med Internet Res. 2024 Aug 6;26:e59005. doi: 10.2196/59005 (PMC11336510; doi:10.2196/59005)
Supplement: Multimedia Appendix 1 [file jmir_v26i1e59005_app1.docx]

## Multimedia Appendix

**Table 1.** Articles related to medical education experiences in Second Life (SL) during undergraduate, residency, and continuing medical education (CME).^a^

| Study | Learners | Description |
| --- | --- | --- |
| Lorenzo-Álvarez et al [33] | Medical students and radiology residents (N=809) | This article provides an overview of the educational opportunities offered by SL for undergraduate and postgraduate radiology training. On the basis of experiences from 2011 to 2018, it highlights how SL facilitates both synchronous and asynchronous learning activities, thus contributing to the development of interpretive and noninterpretive skills. |
| Wiecha et al [45] | Primary care physicians (N=14) | A pilot course on type 2 diabetes was conducted to explore the use of SL for CME and evaluate its effectiveness. The results suggest that the use of virtual worlds can improve learning outcomes beyond what traditional online or face-to-face methods for continuing professional development offer. |
| Melús-Palazón et al [46] | Primary care physicians (N=76) | Qualitative analysis of clinical sessions conducted in SL as part of a CME program in primary care, which found SL to be an effective tool for accessing medical education without the need for physical travel, although recognizing that there are technical challenges that must be addressed. |
| Schwaab et al [62] | Emergency medicine residents (N=27) | Prospective observational study in which residents who performed an in-person mock oral exam and a similar simulated oral exam in SL found the virtual activity realistic (92.6%), concluding that virtual simulation in SL is a possible alternative for simulated oral exams. |
| Richardson et al [63] | Medical students (—^b^) | Descriptive article that explores the use of SL for teaching anatomy in higher education, reviewing previous experiences. The authors conclude that SL allows students to explore 3D models and participate in interactive discussions, improving their conceptual understanding and facilitating inquiry-based learning. |
| Gazave and Hatcher [64] | Medical students (N=39) | Original study to evaluate TBL^c^ in SL to improve teamwork and critical thinking skills in an online anatomy course. In total, 95% of the students agreed that it was a worthwhile experience. The authors propose that virtual TBL sessions are valuable and could be implemented in other online courses. |
| Lorenzo-Álvarez et al [65] | Medical students (N=156) | A randomized controlled trial to compare the effectiveness of practical radiology learning by medical students in SL versus the real world. Since no differences were found in the pre- and postexposure tests, the study concluded that virtual worlds allow for learning x-ray interpretation skills with similar success to that conventional in-person activities. |
| Lorenzo-Álvarez et al [66] | Medical students (N=46) | Pilot study exploring the application of SL to undergraduate radiology teaching through a 4-week voluntary program based on synchronous sessions and asynchronous assignments. In conclusion, SL offered effective and engaging radiology education for medical students, with potential improvements in collaborative learning and practical skills. |
| Lorenzo-Álvarez et al [67] | Medical students (n=48) and family physicians (n=14) | Original study on the perception and attitudes of medical students and family doctors regarding learning radiology in SL through a 3-week course. The experience generated positive opinions and attitudes in undergraduate and graduate attendees, minimized travel costs, and provided background knowledge for subsequent projects on radiology learning in SL. |
| Pino-Postigo et al [68] | Radiology residents (N=67) | Original study on an online course carried out in SL to improve the radiological interpretation skills of residents. These types of online activities based on the interpretation of clinical cases are ideal for the training of residents through dynamic and participatory sessions using high-quality audio and chat. |
| Alonso-Martínez et al [69] | Medical students (n=154) and radiology professors (n=9) | Original study on the perception of teachers without experience in SL after giving a 1-hour lecture and of the students who received it, which revealed that virtual lectures in SL are highly enriching as an experience of professional growth for students and training of trainers, reducing costs and travel times. |
| Rudolphi-Solero et al [70] | Medical teachers (N=23) | Study on the perception of medical teachers regarding the educational possibilities of SL after a 3-hour session within the virtual world. All participants found SL useful and interesting as an educational platform, and 44% were willing to carry out teaching activities in SL. |
| Rampling et al [71] | Medical students (N=24) | Original study consisting of the design of a virtual patient with psychosis in SL for problem-based learning in psychiatry. Only 24 of 150 students participated, whose feedback was predominantly negative. Students expressed that the scenario was cumbersome, did not imitate real life, and had little educational value. |
| Jivram et al [72] | Medical students (N=244) | This study addressed the use of SL to improve problem-based learning in medical education comparing it with interactive web-based methods. Although a minority of students reported that the SL experience seemed more realistic, most preferred web-based methods due to their simplicity and effectiveness. |
| Mitchell et al [73] | Primary care physicians (N=13) | Pilot study in which a motivational interviewing training program was designed and tested in SL to counsel patients about colorectal cancer screening, evaluating training effectiveness, acceptability of the SL environment, and instructional design. Results suggest that virtual worlds offer potential to improve patient-centered communication skill training. |
| Pino-Postigo et al [74] | Radiology residents (N=23) | This study aimed to conduct a 4-week meeting in SL to improve the oral presentation skills of radiology residents and evaluate their perception. In conclusion, SL can be used effectively to train oral communication skills in public through an interesting and useful experience highlighting social contact with peers. |
| McGrath et al [75] | Emergency medicine residents (N=35) | Residents were randomly assigned to a traditional oral examination format (n=17) or a virtual examination format (n=18) conducted in SL. Both groups scored without significant differences and thought that their assessment was realistic, fair, objective, and efficient, but examinees in the virtual group found it a less intimidating format. |
| Danforth [76] | Medical students (—) | Descriptive article focused on the development of virtual patient simulations for medical education through interaction in SL. The authors propose that students can engage in a conversation in natural language with the simulated patient to obtain relevant clinical information, develop differential diagnoses, and propose appropriate treatments. |
| Toro-Troconis [77] | Medical students (N=56) | Doctoral thesis on the design and development of a virtual platform for respiratory medicine patients using a gamification approach in SL. Similar attitudes were observed in the SL group and in the group that used an interactive electronic module. Women showed a more positive attitude toward the perceived usefulness of virtual patients. The repetitive linear presentation of cases was not sufficiently motivating, so the use of more challenging branching learning experiences is recommended for virtual patient delivery. |
| Andrade et al [78] | Geriatric medicine fellows (N=8) | The authors developed a simulation in SL with security risks in an older person’s home included as a virtual station in a 16-station OSCE^d^. A total of 6 participants (75%) rated the simulation as “excellent.” Avatar-based virtual home security OSCE is a practical and acceptable alternative to traditional OSCE stations. |
| Kava et al [79] | Urology residents (N=12) | This study evaluated the feasibility of 2 OSCE environments in SL that allow for the assessment of residents’ communication skills. The method was feasible, acceptable, and applicable to evaluate communication skills. Technical improvements are needed to improve nonverbal cues, focus on individual skills, and provide immediate feedback. |
| Pérez Baena et al [80] | Medical students (N=180) | Gamification study in OSCE virtual scenarios of radiology in SL that aimed to evaluate the educational impact of TBL and students’ perception. The experience, feasible and reproducible, promoted clinical reasoning and teamwork among students in a playful context that they recognized and highly valued. |
| Lorenzo-Álvarez et al [81] | Medical students (N=91) | Original study in which a 6-week competitive learning game about radiology in SL was designed. Experience demonstrated that competitive game-based learning in SL is an effective method for teaching radiology to medical students. Medium-term knowledge results indicated effective learning, and positive perceptions were observed even among nonparticipants. |
| Rudolphi-Solero et al [82] | Medical students (N=373) | Original study to evaluate the impact of mandatory participation in a competitive game in SL to learn undergraduate radiology. In total, 2 consecutive annual editions were analyzed and compared with a previous voluntary edition, concluding that voluntary participation is preferable to maintain the motivation and commitment of students. |
| Rudolphi-Solero et al [83] | Medical students (N=52) | Study to evaluate the adaptation to team competition of a multiuser game to learn radiology in SL. Most participants recognized working as a team and that competitive games helped them learn better. The improved academic and postexposure results compared to those of nonparticipants indicated the potential impact of the game on learning. |
| Rudolphi-Solero et al [84] | Medical students (N=300) | Original study that aimed to evaluate an interuniversity competition to learn radiology held in SL for 2 consecutive years. Participants found the team game useful to reinforce their radiology knowledge and identified this activity as a playful learning and social interaction experience during the COVID-19 pandemic. |
| Danforth [85] | Medical students (—) | This article describes the design of an interactive 3D virtual model of the human testis, highlighting its potential to enhance undergraduate anatomy medical education through immersive learning environments and visualization of complex physiological concepts such as spermatogenesis. |
| Richardson-Hatcher et al [86] | Medical students (—) | Descriptive article on the use of the SL platform to teach the anatomy of the pterygopalatine fossa. The authors created interactive anatomical models, including a basic cone model and an organ model, to improve understanding of this complex region and the nerves that run through it. |
| Alonso-Martínez et al [87] | Medical students (N=174) | Qualitative study on the use of social games (Trivial and The Alphabet) to learn radiology in SL. In conclusion, social games such as television quizzes conducted in SL are effective in connecting students to radiology knowledge in a dynamic and fun way and can be a valuable complement to traditional teaching. |
| Ravaei et al [88] | Medical students (N=157) | This study examined the use of SL to teach undergraduate radiology during the COVID-19 lockdown, presenting the benefits and disadvantages. The authors highlighted the easy adaptation of the students to the 3D platform despite the technical limitations and the very high scores of the experiences. |

^a^Studies related to the training of other professions such as nursing, psychology, and veterinary medicine were outside the focus of this review.

^b^Not applicable. The article did not specify a number of learners.

^c^TBL: team-based learning.

^d^OSCE: objective structured clinical examination.
